# Hepatolenticular degeneration-induced hepatic dysfunction with extremely atypical clinical manifestations: a Case Report

**DOI:** 10.3389/fmed.2025.1599283

**Published:** 2025-05-14

**Authors:** Zhuang Tao, Jiafeng Zhou, Zhenzhen Jiang, Ya Hu, Shupei Jia, Meixia Wang

**Affiliations:** ^1^Encephalopathy Center, The First Affiliated Hospital of Anhui University of Chinese Medicine, Hefei, China; ^2^Graduate School, Anhui University of Chinese Medicine, Hefei, China

**Keywords:** hepatolenticular degeneration, hepatic dysfunction, atypical clinical manifestations, case report, whole exome sequencing

## Abstract

Hepatolenticular Degeneration (HLD) is a rare condition caused by a genetic copper metabolism disorder and a basal ganglia-dominated degenerative brain disease. Its characteristic clinical features include progressive extrapyramidal symptoms, psychiatric manifestations, cirrhosis, renal impairment, and the Kayser-Fleischer ring. Furthermore, its key diagnostic bases include the ceruloplasmin level, copper oxidase activity, trace copper in the human body, brain Magnetic Resonance Imaging (MRI), and genetic testing. Here, we present an HLD case with atypical clinical manifestations. A 43-year-old male HLD patient presented to our hospital with normal copper oxidase activity and serum copper levels, as well as results of ceruloplasmin testing, slit-lamp examination, and histopathological examination of the liver, which showed no typical manifestations. On the other hand, the genetic testing results showed new mutation sites. To improve our clinical understanding of HLD and reduce the probability of misdiagnosis and missed diagnosis, we discussed and clarified the clinical manifestations, pathogenesis, and diagnosis and treatment of the disease, all based on existing literature.

## 1 Introduction

Hepatolenticular Degeneration (HLD) is a copper metabolism disorder caused by mutations in the ATPase Copper Transporting Beta (ATP7B) gene that manifests primarily as liver injury and neuropsychiatric symptoms (1). Epidemiological data suggest that the disease's incidence, prevalence, and frequency of carriers are 15–30/million people across different populations, ~1/30,000 people, and 1 in 90, respectively. Furthermore, although the illness can occur at any age, it mostly affects individuals aged 5–35 years (2, 3). Since excess copper cannot be excreted, it is often deposited in various organs and tissues including the liver, cranium, kidney, cornea, and bone joints, potentially resulting in various symptoms. Additionally, different organs might exhibit varying degrees of involvement and the clinical manifestations might be diverse and non-specific, further increasing the risk of misdiagnosis or missed diagnosis (4, 5).

Here, we present an HLD case with atypical clinical manifestations. The patient showed normal copper oxidase activity and serum copper levels, as well as results of ceruloplasmin testing, slit-lamp examination, and histopathological examination of the liver which showed no typical symptoms. However, the genetic testing results showed new mutation sites. To improve our clinical understanding of HLD and reduce the probability of misdiagnosis and missed diagnosis, we discussed the clinical manifestations, pathogenesis, and diagnosis and treatment of the disease, all based on existing literature.

## 2 Case presentation

### 2.1 Present history

A 43-year-old male patient presented to our hospital on November 27, 2024, with “a 4-year history of Hepatic Dysfunction (HD).” On April 28, 2020, the patient was examined at the Taizhou Enze Medical Center (Group) and exhibited elevated levels of Alanine Aminotransferase (ALT; 84 U/L↑), Aspartate Aminotransferase (AST; 54 U/L↑), γ-Glutamyl Transpeptidase (GGT; 88 U/L↑), α-L-fucosidase (AFU; 84 U/L↑), and Alpha-Fetoprotein (AFP; 10.3 ng/ml↑). Furthermore, the ultrasound examination of the liver, gallbladder, pancreas, and spleen indicated a fatty liver, instigating lifestyle adjustments, albeit without proper attention and diagnosis.

During a physical examination at a local hospital in September 2022 (no report available), the patient showed abnormal liver function indexes. Consequently, on September 27, 2022, the patient was admitted to the Third Affiliated Hospital of the Naval Medical University (Yangpu Campus) for a Magnetic Resonance (MR) enhancement of the liver, revealing cirrhosis, multiple sclerotic nodules, parapancreatic lymph nodes, fatty liver, and cholecystitis. The patient was then put on oral hepatoprotective drugs (details unknown).

The patient later visited the Taizhou Enze Medical Center (Group) as well as the Third Affiliated Hospital of the Naval Medical University (Yangpu Campus) several times between October 9, 2022 and April 25, 2023, where he underwent several tests. According to the results, the patient exhibited elevated levels of ALT (74–93 U/L↑), AST (38–56 U/L↑), GGT (85–119 U/L↑), AFT (16.10–19.53 ng/ml↑), AFU (89–105 U/L↑). The patient showed no significant abnormalities in the Hepatitis B five-panel test; Liver diseases—related autoantibodies (AMA-M2, LKM-1, SLA/LP, LC-1), and Systemic rheumatic disease autoantibodies (SSA, SSB, Jo-1, RNP, Sm, Scl-70, ACA, dsDNA, NA, HA, RPPA, PCNA, PM-Scl). Based on these results, the patient was intermittently put on oral hepatoprotective drugs [including, ursodeoxycholic acid capsules (250 mg oral tid), polyene phosphatidylcholine capsules (0.456 mg oral tid), and vitamin E (0.1 g oral bid), among other medications].

On June 19, 2023, the patient underwent several tests at Renji Hospital, Medical College of Shanghai Jiao Tong University. The results showed reduced ceruloplasmin levels (0.13 g/L↓). Furthermore, genetic testing revealed the existence of two heterozygous missense variants in the ATP gene: c.1571T > C (p.Met524Thr), and c.3809A > G (Asn1270Ser; Reported on 2023-07-13). Based on these results, the patient was diagnosed with HLD and prescribed ursodeoxycholic acid capsules (250 mg oral tid), polyene phosphatidylcholine capsules (0.456 mg oral tid), vitamin E tablets (0.1 g oral bid), and zinc gluconate tablets (350 mg oral tid). During follow-up examination between September 25, 2023 and November 29, 2023, the patient showed improvement in the liver function indicators, although the ALT (56–58 U/L↑), AST (34–43 U/L↑), GGT (64–69 U/L↑), AFU (60.60–68.20 U/L↑), and AFT (11.10 ng/ml↑) levels remained relatively high. Furthermore, ceruloplasmin levels (0.17–0.19 g/L) had improved. It is also noteworthy that the liver, gallbladder, pancreas, spleen, and liver ultrasound-elastography quantification revealed mild fatty liver with multiple hypoechoic nodules, a slightly enlarged spleen, and no apparent abnormalities in the gallbladder and pancreas. Both the mean and median liver elasticity measurement values were 8.4 kPa.

From Nov 28, 2023, to Apr 01, 2024, the patient was hospitalized three times at the Second Affiliated Hospital of Medical College of Zhejiang University. The ceruloplasmin levels ranged between 0.15 g/L and 0.16 g/L. Furthermore, the ultrasonography of the liver, gallbladder, pancreas, and spleen revealed changes in liver echogenicity, which were consistent with the HLD ultrasonography results that showed a fatty liver with multiple hypoechoic nodules, examinations were recommended to further determine the patient's condition. Furthermore, ultrasound-elastography examination revealed liver stiffness (19.24 Kpa) and whole abdominal CT enhancement revealed cirrhosis, a slightly enlarged spleen, portal Hypertension (HTN), and varicose veins around the fundus of the stomach. Calcified foci were also observed in segment VIII of the liver, along with adenomyosis at the base of the gallbladder. Moreover, liver MRI with Diffuse-Weighted Imaging (DWI) revealed cirrhosis with diffuse intrahepatic regenerative nodules and splenomegaly. Other findings included gallbladder adenomyosis with chronic atrophic cholecystitis. Based on these results, the patient was prescribed sodium dimercaptopropanesulfonate, compound glycyrrhizin, glutathione, zinc gluconate, vitamin C, and vitamin E tablets. After discharge, the patient was put on irregular doses of vitamin C, zinc gluconate, penicillamine, vitamin B6, methylcobalamin, and fursutiamine tablets.

**Figure 1:**
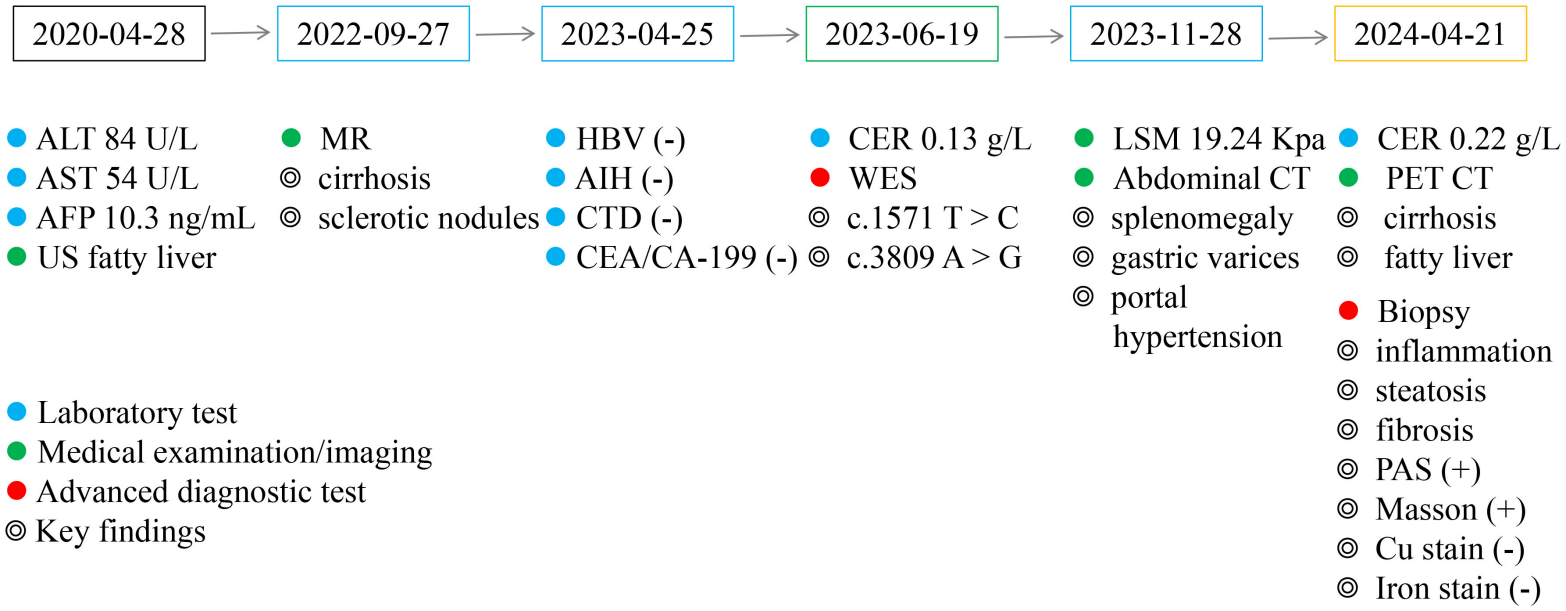
Key findings of patient at critical time points. ALT, alanine aminotransferase; AST, aspartate aminotransferase; AFP, alpha-fetoprotein; MR, magnetic resonance; HBV, hepatitis B virus serological markers; AIH, autoimmune hepatitis; CTD, connective tissue diseases; CEA, carcinoembryonic antigen; CA19-9, carbohydrate antigen 19-9; CER, ceruloplasmin; WES, whole exome sequencing; LSM, liver stiffness measurement. The figure shows key findings at patient's critical time points.

In the same period, the patient underwent several tests across multiple visits at Shulan Hospital due to multiple hypoechoic nodules in the liver. The ceruloplasmin levels were at 0.22 and 0.18 g/L at 2024.01.03 and 2024.04.21, respectively. On the other hand, whole-body PET/CT revealed cirrhosis, a fatty liver, and no apparent abnormal density and hypermetabolic foci in the liver parenchyma. The liver, gallbladder, pancreas, and spleen were also examined revealing findings suggestive of cirrhosis, splenomegaly, a fatty liver, and multiple intrahepatic hypoechoes. On April 29, 2024, the patient underwent percutaneous liver biopsy of the right hepatic parenchyma. The pathological results indicated the presence of a hepatic lobular structure, regular hepatic trabeculae arrangement, turbid and swollen liver cells, visible focal necrosis, mild mixed liver steatosis, fibrous tissue proliferation with a fibrous septal formation tendency and a chronic inflammatory cell infiltration in the portal areas, and mild interfacial inflammation. Based on these results, the patient was diagnosed with mild chronic hepatitis (G2S2-3), accompanied by mild steatosis. The patient also underwent several histochemical staining tests and the results obtained were as follows: Copper (-), D-PAS (-), PAS (+), Trichrome Masson (+), Iron (-), and reticulin (mesh) (+). The Immunohistochemistry (IHC) results were: C4d (-), CD10 (capillary duct+), CD4 [(hepatic sinusoids + lymphocytes) +], CD8 (lymphocytes +), CK19 (bile ducts +), CK7 (bile ducts +), hepatocytes (+), KI67 (3%+), HBSAG (-), and GS (+). Main details regarding the present history are shown in Table 1 and Figure 1.

**Table 1:** Main details of the patient's present history.

**Date**	**Hospital**	**Main reports**	**Interventions**
2020-04-28	Taizhou Enze Medical Center (Group)	ALT 84 U/L, AST 54 U/L, GGT 88 U/L, AFU 84 U/L, AFP 10.3 ng/ml; Abdomen ultrasound (US), a fatty liver	Lifestyle adjustments
2022-09-27	The Third Affiliated Hospital of the Naval Medical University (Yangpu Campus)	AFP 17.0 ug/L, AFP-L3 (-); Magnetic resonance imaging (MRI), cirrhosis, multiple sclerotic nodules, parapancreatic lymph nodes, fatty liver, and cholecystitis	Oral hepatoprotective drugs (details unknown)
2022-10-09	Taizhou Enze Medical Center (Group)	ALT 93 U/L, AST 56 U/L, GGT 119 U/L, AFP 16.1 ng/ml	Ursodeoxycholic acid capsules (250 mg oral tid), polyene phosphatidylcholine capsules (0.456 mg oral tid), and vitamin E (0.1 g oral bid)
2022-11-10	Taizhou Enze Medical Center (Group)	AFP 19.53 ng/ml, Hepatitis B Virus Serological Markers (-)	
2022-11-22	The Third Affiliated Hospital of the Naval Medical University (Yangpu Campus)	ALT 74 U/L, AST 38 U/L, GGT 89 U/L, AFU 105 U/L, AFP 17.1 ng/ml, AFP-L3 (-), CEA, CA19-9 (-); Liver diseases - related autoantibodies, AMA-M2, LKM-1, SLA/LP, LC-1, (-); MRI, cirrhosis, multiple sclerotic nodules, parapancreatic lymph nodes, fatty liver, and cholecystitis	
2023-04-25	The Third Affiliated Hospital of the Naval Medical University (Yangpu Campus)	ALT 81 U/L, AST 46 U/L, GGT 85 U/L, AFU 89 U/L; AFP 17.5 ug/L, AFP-L3% 28.1 %, AFP-L3 (+); Systemic rheumatic disease autoantibodies SSA, SSB, Jo-1, RNP, Sm, Scl-70, ACA, dsDNA, NA, HA, RPPA, PCNA, PM-Scl (-); MR revealed cirrhosis, multiple sclerotic nodules, parapancreatic lymph nodes, fatty liver, and cholecystitis	
2023-06-19	Renji Hospital, Medical College of Shanghai Jiao Tong University	ALT 50 U/L, AST 61 U/L, GGT 73 U/L, AFU 74.40 U/L; CER 0.13 g/L	
2023-07-13	Shanghai Children's Medical Center Affiliated to Shanghai Jiao Tong University of Medicine	WES two heterozygous missense variants in the ATP gene: c.1571T > C (p.Met524Thr), and c.3809A > G (Asn1270Ser)	Ursodeoxycholic acid capsules (250 mg oral tid), polyene phosphatidylcholine capsules (0.456 mg oral tid), vitamin E tablets (0.1 g oral bid), and zinc gluconate tablets (350 mg oral tid)
2023-10-24	Shanghai Children's Medical Center Affiliated to Shanghai Jiao Tong University of Medicine	WES (Patient's sister) two heterozygous missense variants in the ATP gene: c.1571T > C (p.Met524Thr), and c.3809A > G (Asn1270Ser); WES (Patient's father) one heterozygous missense variant in the ATP gene: c.3809A > G (Asn1270Ser)	
2023-11-29	Renji Hospital, Medical College of Shanghai Jiao Tong University	ALT 58 U/L, AST 43 U/L, GGT 64 U/L, AFU 68.20 U/L; AFP 11.1 ng/mL; CER 0.19 g/L	
2023-12-15	The Second Affiliated Hospital of Medical College of Zhejiang University	CER 0.15 g/L; ALT 57 U/L; CIV 51.21 ng/ml; Abdomen US a fatty liver with multiple hypoechoic nodules, LSM (19.24 Kpa); Abdominal CT, cirrhosis, a slightly enlarged spleen, portal Hypertension, and varicose veins around the fundus of the stomach MRI, cirrhosis with diffuse intrahepatic regenerative nodules and splenomegaly. Other findings included gallbladder adenomyosis with chronic atrophic cholecystitis	Sodium dimercaptopropanesulfonate, compound glycyrrhizin, glutathione, zinc gluconate, vitamin C, and vitamin E tablets.
2025-04-21	Shulan Hospital	ALT 69 U/L, AST 43 U/L, AFU 59 U/L,AFP 8.9 ng/mL; CER 0.22 g/L; Abdomen US, cirrhosis, a fatty liver, and multiple intrahepatic hypoechoes, LSM 22.9 KPa; PET/CT, cirrhosis, a fatty liver, and no apparent abnormal density and hypermetabolic foci in the liver parenchyma. It also revealed ground-glass nodules in the upper lobe of the right lung, and small nodules in the lower lobe of the right lung, which appeared benign. Furthermore, there were findings suggestive of hyperplastic lymph nodes in the right cardiophrenic angle, mediastinum, and right armpit, along with signs of esophagitis and duodenal diverticulum. The PET/CT results further revealed potential cervical 6/7 vertebral endplate inflammation, possibly due to spinal degenerative changes	Ursodeoxycholic acid capsules, Rabeprazole Sodium Enteric-coated Capsules, Teprenone Capsules
2024-05-07	Shulan Hospital	biopsy of the right hepatic parenchyma, a hepatic lobular structure, regular hepatic trabeculae arrangement, turbid and swollen liver cells, visible focal necrosis, mild mixed liver steatosis, fibrous tissue proliferation with a fibrous septal formation tendency and a chronic inflammatory cell infiltration in the portal areas, and mild interfacial inflammation. Based on these results, the patient was diagnosed with mild chronic hepatitis (G2S2-3), accompanied by mild steatosis. The patient also underwent several histochemical staining tests and the results obtained were as follows: Copper (-), D-PAS (-), PAS (+), Trichrome Masson (+), Iron (-), and reticulin (mesh) (+). The Immunohistochemistry (IHC) results were: C4d (-), CD10 (capillary duct+), CD4 [(hepatic sinusoids + lymphocytes) +], CD8 (lymphocytes +), CK19 (bile ducts +), CK7 (bile ducts +), hepatocytes (+), KI67 (3%+), HBSAG (-), and GS (+)	

ALT, alanine aminotransferase; AST, aspartate aminotransferase; GGT, γ-glutamyl transpeptidase; AFU, α-L-fucosidase; AFP, alpha-fetoprotein; AMA-M2, anti-mitochondrial antibody type 2; LKM-1, liver-kidney microsomal-1; SLA/LP, soluble liver antigen/liver-pancreas; LC-1, liver cytosol antibody-1; SSA, sjögren's syndrome A; SSB, sjögren's syndrome B; RNP, ribonucleoprotein; Sm, Smith antibody; Scl-70, topoisomerase I; ACA, anti-centromere antibody; dsDNA, double-stranded DNA; NA, nucleosome antibody; HA, histone antibody; RPPA, ribosomal P protein antibody; PCNA, proliferating cell nuclear antigen; PM-Scl, polymyositis-scleroderma; CEA, carcinoembryonic antigen; CA19-9, carbohydrate antigen 19-9; CER, ceruloplasmin; WES, whole exome sequencing; CIV, type IV collagen; LSM, liver stiffness measurement.

**Past history: (-)**

**Personal history: (-)**

### 2.2 Family history

The patient was born to consanguineous parents. On 2023-06-20, the patient's sister underwent genetic testing at Shanghai Children's Medical Center, which is affiliated to the Medical College of Shanghai Jiao Tong University, revealing two heterozygous missense variants in the ATP gene: c.1571T > C (p.Met524Thr) and c.3809A > G (Asn1270Ser). The patient's father underwent similar tests on 2023-10-24, revealing the presence of one heterozygous missense variant in the ATP gene: c.3809A > G (Asn1270Ser). There were no genetic testing results for the mother as she had died prematurely. Notably, the patient's sister was later diagnosed definitively with HLD on November 28, 2023.

**Figure 2:**
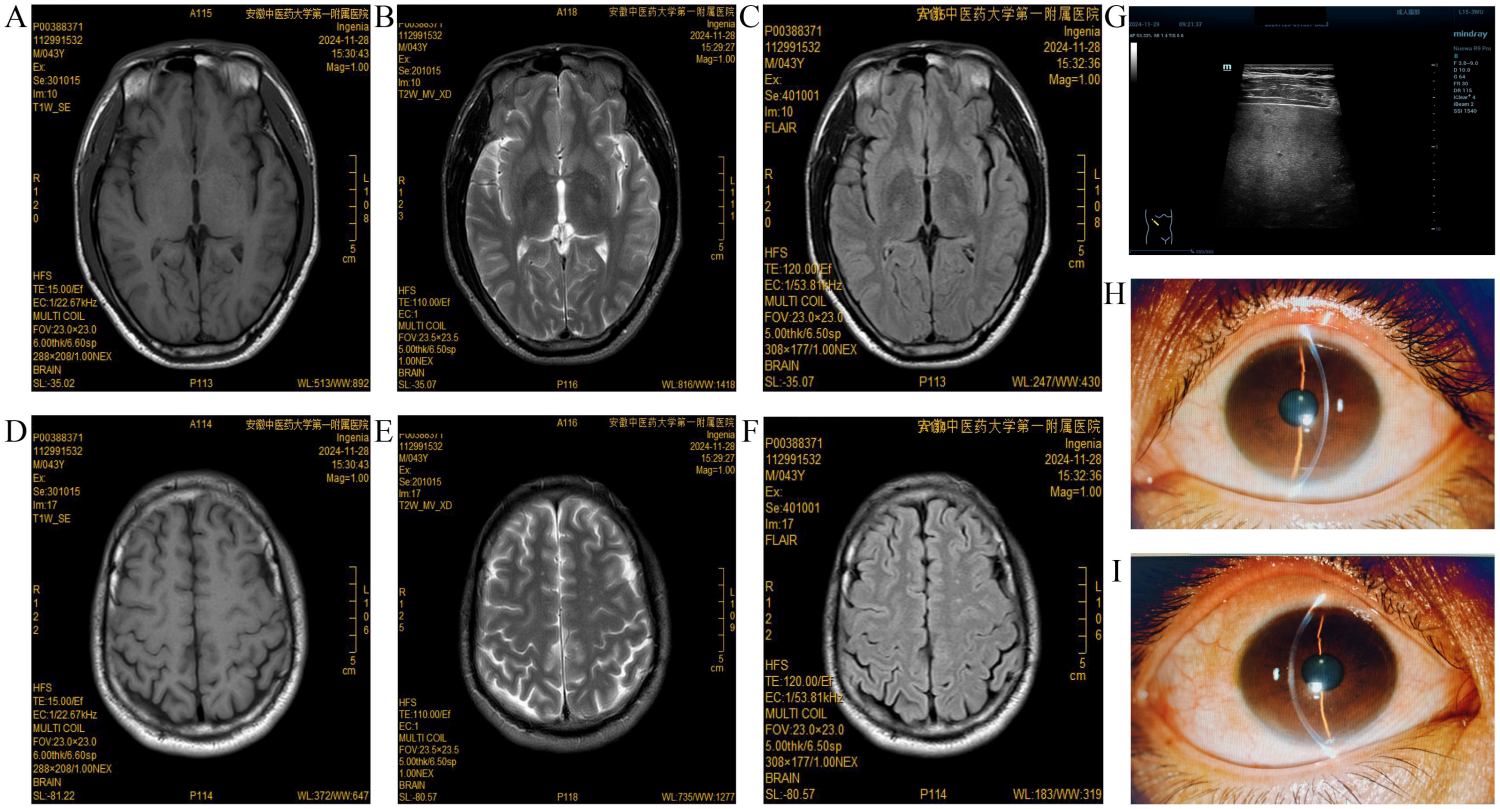
Brain MRI, liver ultrasonography and corneal K-F ring examination. **(A–C)** T1, T2, and Flair high signal in the left lentiform nucleus on the brain MRI plain scan; **(D–F)** Multiple speckled T1WI or slightly lower T2WI high signal shadows, with a high signal on FLAIR in the bilateral cerebral cortex on brain MRI plain scan; **(G)** Multiple hyperechoic nodules scattered in the liver; and **(H, I)** Few corneal K-F rings observed during slit-lamp examination.

Supplementary tests were further conducted revealing a ceruloplasmin level of 0.211 g/L and a copper oxidase activity of 0.395 units. Serum di-element analysis revealed copper at 11.52 μmol/L and zinc at 16.01 μmol/L. Furthermore, 24 h urinary elements were examined revealing copper at 1,475.11 μg/24 h↑ and zinc at 2,815.86 μg/24 h↑. On the other hand, the results of liver function, kidney function, electrolytes, blood glucose, and lipid tests were ALT at 78.3 U/L↑, collagen type IV at 107.565 ng/mL↑, and hyaluronic acid at 276.277 ng/mL↑. Results consistent with Coombs negative hemolysis were also obtained. No significant abnormalities were observed in routine blood tests, immune panel, coagulation profile, urinalysis, fecal examination including occult blood, and electrocardiogram tests. Regarding Chest CT, there were a few old foci in both lungs and small ground-glass nodules in the right lung. Additionally, renal ureteral bladder ultrasonography revealed crystallization in both kidneys and the abdominal ultrasonography of the liver, gallbladder, pancreas, and spleen revealed dense echoes in the liver with uneven distribution and larger multiple hyperechoic nodules scattered in the liver (16 mm × 11 mm). There was also a possibility of uneven fat deposition in the liver parenchyma and liver island changes. The median values of five consecutive measurements on liver echo-elastography were: shear wave velocity (Vs) = 2.46 m/s, liver stiffness (E) = 18.34 Kpa, and liver fat content (UDFF) = 7 %. Finally, the brain MRI scan revealed few subcortical foci on the bilateral cerebral cortex, slit-lamp examination showed positive corneal K-F rings, and the Leipzig score was 9 points. Details regarding the Brain MRI, liver ultrasonography and corneal K-F ring examination are shown in Figure 2.

### 2.3 Diagnosis

Based on the aforementioned findings, the patient was diagnosed with HLD.

### 2.4 Treatment and follow-up

The intervention encompassed a low-copper diet, a sodium dimercaptopropane sulfonate injection (20 mg/kg/d), a glutathione injection (1.8 g intravenously infused qd), zinc gluconate tablets (350 mg oral tid), and alfacalcitol soft capsules (0.25 μg oral qd).

Liver function re-examination (December 9, 2024): the results were ALT at 87.6 U/L, AST at 54.7 U/L, and GGT at 65 U/L. Others were collagen type IV at 94.119 ng/mL, hyaluronic acid at 334.550 ng/mL↑, and laminin at 130.774 ng/mL↑. For lipids, the total cholesterol level was 5.93 mmol/L↑ and the 24-h urine analysis revealed copper at 1,151.00 μg/24 h↑, zinc at 2,462.83 μg/24 h↑, and calcium at 78.28 mg/24h↓.

The patient was discharged from the hospital with a prescription of regular doses of dimercaptosuccinic acid capsules (0.5 g oral bid), glutathione tablets (0.2 g oral tid), zinc gluconate tablets (350 mg oral tid), and alfacalcitol soft capsules (0.25 μg oral qd).

Further examinations were conducted on February 3, 2025, revealing ceruloplasmin levels at 0.199 g/L and copper oxidase activity at 0.281 units. The analysis of serum di-elements revealed copper at 10.43 μmol/L↓ and zinc at 4.67 μmol/L↓. Additionally, 24 h urine analysis revealed copper at 1,263.78 μg/24 h↑ and zinc at 2,418.69 μg/24 h↑. Furthermore, the liver function, kidney function, electrolytes, blood glucose, and lipid tests revealed ALT at 65.7 U/L↑ and AST at 44.7 U/L. Finally, regarding the hepatofibrillar elements, collagen type IV and hyaluronic acid were detected at 97.724 ng/mL↑ and 260.345 ng/mL↑, respectively.

## 3 Discussion

Our patient's primary clinical manifestation was HD, with K-F rings visible in the cornea. He also exhibited elevated 24-h urine copper levels and decreased ceruloplasmin levels. Furthermore, genetic testing revealed the presence of heterozygous mutations in the ATP7B gene, leading to a definitive HLD diagnosis. Moreover, several disturbing factors were noted in the patient's diagnosis and treatment. First, the serum ceruloplasmin level did not decrease significantly (with one measurement even falling within a normal range) and the copper oxidase activity was normal. Second, the serum copper level remained normal even after several checks. Finally, liver copper staining revealed negative results.

The patient was subjected to genetic testing, revealing the presence of two heterozygous missense variants in the ATP gene: c.1571T > C (p.Met524Thr) and c.3809A > G (Asn1270Ser). Notably, the 3809 site is located in the highly conserved phosphorylation region of the ATP7B protein, and 3,809 mutation affects ATP7B protein phosphorylation; hence, the localization of ATP7B protein in the cell is not dependent on copper ion concentration (6). According to the classification standard and guidelines of ACMG mutation, c.3809A > G (Asn1270Ser), one of the common hotspot mutations in the ATP7B gene, can be classified as a “pathogenic mutation” (7, 8). It is also noteworthy that the 1,571 site in exon 4, a copper ion binding region, is involved in the coding of copper ion activation and ceruloplasmin synthesis. On the other hand, c.1571T > C (p.Met524Thr) is a newly discovered mutation and its pathogenicity is still unclear; hence, it is tentatively categorized as a “mutation with uncertain significance” (9). These two sites have a very low mutation frequency, and the lack of relevant functional experimental studies and case reports makes it difficult to explain their correlation with the clinical manifestations of this case.

**Figure 3:**
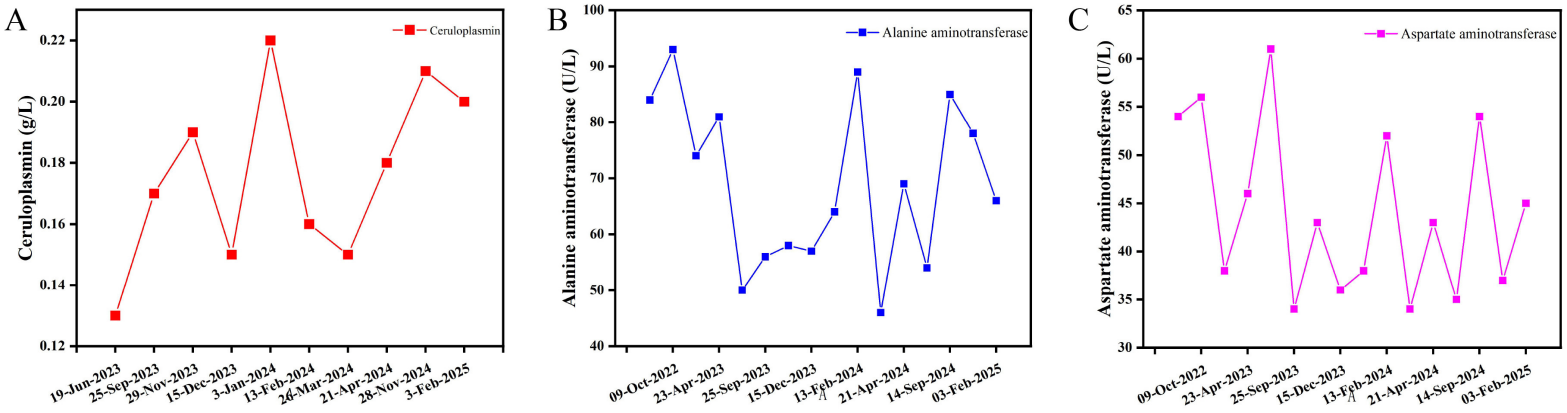
Monitoring charts for the serum ceruloplasmin level and liver function indexes. **(A)** Serum ceruloplasmin level monitoring chart; **(B)** Alanine aminotransferase monitoring chart; **(C)** Aspartate aminotransferase monitoring chart. Serum ceruloplasmin levels of the patient were slightly low or normal. After diagnosed with HLD in June 19, 2023 and received treatment with copper chelation and liver protection drugs, the patient showed improvement in the liver function indicators between September 25, 2023 and November 29, 2023.

Serum copper oxidase activity indirectly reflects serum ceruloplasmin levels, one of the key diagnostic bases of HLD. According to research, serum copper oxidase activity and ceruloplasmin levels are not related to the disease status and duration and effect of copper removal treatment (10). Normal serum ceruloplasmin levels range between 200 and 400 mg/L, with < 100 mg/L strongly supporting a HLD diagnosis. Furthermore, some HLD patients and ATP7B gene heterozygous mutation carriers might exhibit levels ranging between 100 and 200 mg/L. It is also noteworthy that about one-third of HLD patients may not show reduced ceruloplasmin levels. Furthermore, compared to patients with liver injury as the predominant issue, HLD patients with neurological disorders as the predominant issue might exhibit significantly lower serum ceruloplasmin levels (11). Moreover, other multiple diseases besides HLD have shown reduced serum ceruloplasmin levels, including liver failure, nephrotic syndrome, malnutrition, acquired copper deficiency, and genetic ceruloplasmin deficiency, among others. It is also noteworthy that ceruloplasmin is an acute temporal reactive protein and acute inflammation and hyperestrogenemia-associated conditions (e.g., pregnancy, estrogen supplementation, and use of certain oral contraceptives) could lead to its upregulation in serum (12, 13). Consistent with previous reports (14), our patient, a 43-year-old middle-aged male, developed the disease relatively late and presented with symptoms of liver damage. Multiple re-examinations revealed normal copper oxide activity and a non-significant decrease in serum ceruloplasmin levels, with one test even showing a normal level. Details regarding monitoring charts for the serum ceruloplasmin level and liver function indexes are shown in Figure 3.

The serum free copper level and 24-h urine copper level are important indicators for selecting HLD treatment regimens and determining treatment efficacy (15). The serum copper level (total copper level in serum) is the sum of ceruloplasmin-bound and non-ceruloplasmin-bound copper. Among them, the bound copper, covalently bound to ceruloplasmin, accounts for ~85%~95% of the body's copper. The remaining 5–15% is in the form of free copper, loosely bound to albumin and other small molecules in the blood. While free copper concentration often increases in HLD patients, it could be as high as ≥ 400 μg/L in most untreated patients, inducing toxicity that are closely related to the progression of the disease and increasing the risk of neurological symptom aggravation resulting from copper-removal treatment (10). Our patient exhibited normal serum copper levels after several tests. Furthermore, compared to the pre-treatment level, the free copper level in serum was significantly lower after treatment (66.87 vs. 35.46 μg/L), although the 24-h urine copper level was not controlled satisfactorily (1,475.11 vs. 1,263.78 μg/24 h), a phenomenon attributable to the patient not adhering to a strict low-copper diet and prescribed medication. Nonetheless, our liver function tests and liver fiber indexes confirmed that the liver protection and copper removal treatments were effective in this patient.

**Figure 4:**
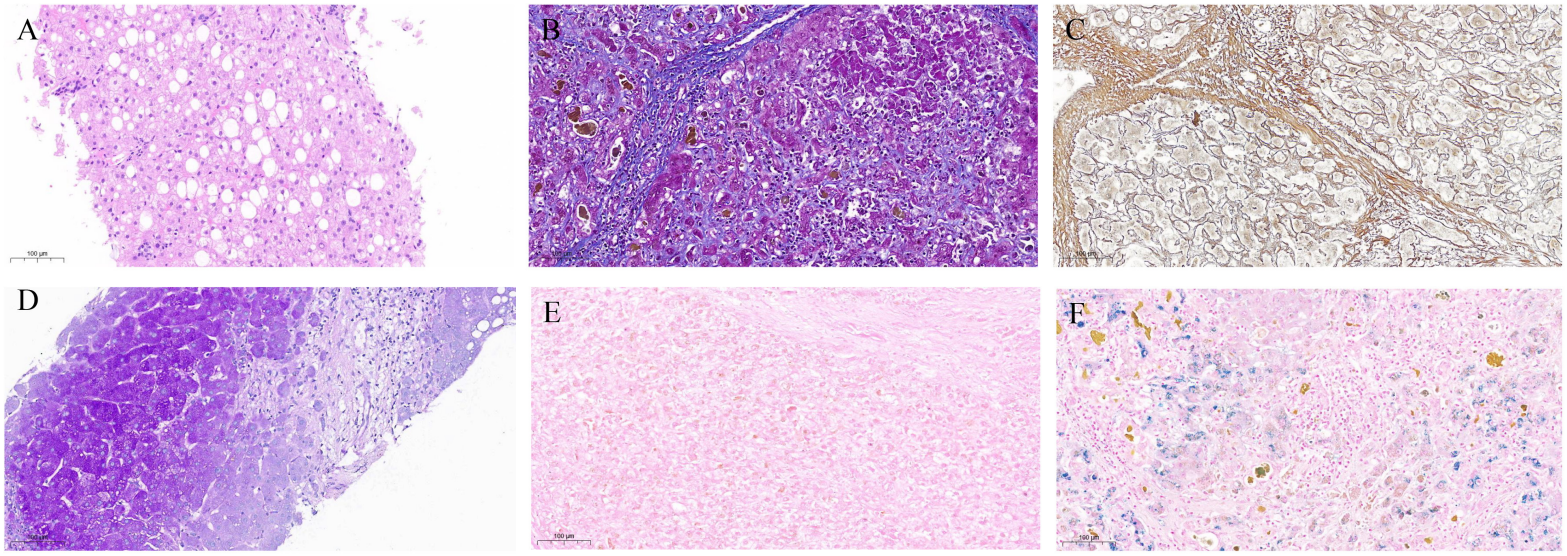
Pathological examination of liver. **(A)** Hematoxylin-Eosin staining, with turbid and swollen liver cells, visible focal necrosis, mild mixed liver steatosis; **(B)** Masson's Trichrome staining (+); **(C)** Reticular Fiber staining (+); **(D)** Periodic Acid-Schiff staining (+); **(E)** Copper staining (−); **(F)** Iron staining (−).

Pathological examination of the liver is useful in histological diagnosis, determining the lesion extent, and assessing treatment efficacy in HLD. Early lesions are often mild and non-specific, exhibiting focal necrosis, apoptotic vesicles, hepatocyte ballooning, hepatocyte steatosis, glycogen-nucleated hepatocytes, and mild inflammation in the portal area. On the other hand, as the disease progresses, lobular inflammatory necrosis appears and confluent zone inflammation intensifies. At this stage, some cases might also present with changes in steatohepatitis or chronic hepatitis. Regarding end-stage HLD, it is noteworthy that some patients might present with macronodular cirrhosis or a mixture of macronodular and micronodular cirrhosis (16). According to research, the inconsistent degree of liver damage, uneven hepatic copper distribution, and discrepancy between pathological and clinical liver function grades in HLD patients might limit the pathological examination of the liver (17, 18). Our patient's histopathological examination results (right hepatic parenchyma) showed turbid swelling of hepatocytes, focal necrosis, mild mixed steatosis, fibroplasia with fibrous septal formation in the portal area, and chronic inflammatory cell infiltration, with mild interfacial inflammation. Others included positive PAS, trichrome Masson, and mesh staining results, while copper staining was negative. Collectively, these results may be related to the uneven distribution of hepatic copper or its low deposition in the early stage of the disease. Details of the pathological examination of liver are shown in Figure 4.

## 4 Conclusion

Overall, our patient's ceruloplasmin levels, copper oxidase activity, serum copper levels, slit-lamp examination results, and liver pathology results were atypical. Therefore, for patients with unexplained HD, a timely sequencing of the full-length coding region of the ATP gene and its flanks, family screening, and 24-h urine copper level examination, are imperative to reduce the likelihood of clinical misdiagnosis and missed diagnosis and the burden of an exacerbated disease.

## Data Availability

The raw data supporting the conclusions of this article will be made available by the authors, without undue reservation.
